# Characterization of resistance genes and plasmids from sick children caused by Salmonella enterica resistance to azithromycin in Shenzhen, China

**DOI:** 10.3389/fcimb.2023.1116172

**Published:** 2023-03-29

**Authors:** Hongmei Wang, Hang Cheng, Baoxing Huang, Xiumei Hu, Yunsheng Chen, Lei Zheng, Liang Yang, Jikui Deng, Qian Wang

**Affiliations:** ^1^ Laboratory Medicine Centre, Nanfang Hospital, Southern Medical University, Guangzhou, China; ^2^ Department of Infectious Diseases, Shenzhen Children’s Hospital, Shenzhen, China; ^3^ School of Medicine, Southern University of Science and Technology, ShenZhen, Guangdong, China; ^4^ Center for Clinical Laboratory, Zhujiang Hospital, Southern Medical University, Guangzhou, China

**Keywords:** azithromycin, *mphA* gene, plasmid, children, *Salmonella* enterica

## Abstract

**Introduction:**

*Samonella* is 1 of 4 key global causes of diarrhoeal diseases, sometimes it can be serious, especially for yong children. Due to the extensive resistance of *salmonella* serotypes to conventional first-line drugs, macrolides (such as azithromycin) have been designated as the most important antibiotics for the treatment of *salmonella*. Antimicrobial resistance is a major public health problem in the world, and the mechanism of azithromycin resistance is rarely studied.

**Methods:**

This study determined the azithromycin resistance and plasmids of *Salmonella* enterica isolates from children attending the Shenzhen Children’s Hospital. The susceptibility of ampicillin (AMP), ciprofloxacin (CIP), ceftriaxone (CRO), sulfamethoxazole (SMZ), chloramphenicol (CL), and azithromycin (AZM) were detected and the genes and plasmids from azithromycin-resistant *Salmonella* were detected by Illumina hi-seq and Nanopore MinIone whole genome sequencing (WGS) using a map-based method, and the genomic background of these factors was evaluated using various bioinformatics tools.

**Results:**

In total, 15 strains of nontyphoid *Salmonella* strains that were isolated (including *S.* typhimurium, *S.*London, *S.* Goldcoast, and *S.*Stanley) demonstrated resistance to azithromycin (minimum inhibitory concentration,MIC from 32 to >256 µg/mL), and the resistance rate was 3.08% (15/487). The sensitivity test to other antibiotics demonstrated 100% resistance to AMP, and the resistance to SMZ and CL was 86.7% and 80.0%, respectively. Through WGS analysis, all isolates were positive for a plasmid-encoded *mphA* gene. Plasmid incompatibility typing identified five *IncFIB(K)*, five *IncHI2/HI2A/Q1*, two *IncC*, one *IncHI2/HI2A/N*, one *IncR*, one *IncFII* and one *IncHI2/HI2A* plasmids. Sequence analyses of plasmids revealed extensive homology to various plasmids or transposons in regions involved in plasmid replication/maintenance functions and/or in antibiotic resistance gene clusters.

**Conclusion:**

*mphA* is the main gene involved in azithromycin, a macrolide, and resistance to *Salmonella*. It is usually located on plasmids and easily spreads, hence posing a great threat to the current treatment of *Salmonella* infection. The plasmid sequence similarities suggest that the plasmids acquired resistance genes from a variety of enterica bacteria and underscore the importance of a further understanding of horizontal gene transfer among enterica bacteria.

## 1 Introduction

*Salmonella* is a gram negative rods belonging to the *Enterobacteriaceae* family and are divided into serotypes according to the structures of H and O antigens on their surface. The two species of *Salmonella* are *S*.bongori and *S.*enterica, *S.*enterica including more than 2,600 serotypes have been shown to be main sources of infections in human. These serogroups include *S.*Typhi,*S.*Paratyphi, *S.*Typhimurium, *S.*Enteritidis, *S.*Choleraesuis, and so on, which can be grouped into typhoidal and nontyphoidal *Salmonella* (NTS) serovars (Gilchrist et al., 2015).

*Samonella* is 1 of 4 key global causes of diarrhoeal diseases, most of salmonellosis is mild, diarrhea, fever and stomach cramps are the main symptoms, people should not take antibiotics and recover in 4 to 7 days (World Health Organization, 2018). Sometimes it can be serious, especially for yong children, it was reported by WHO that 550 million people falling ill and 220 million chidlren under the age of 5-year-old each year (World Health Organization, 2018). Current recommendations are that antibiotics be reserved for patients with severe disease or patients who are at a high risk for invasive disease (Guarino et al., 2014). Children with suspected or confirmed invasive infections, including infants younger than three months of age with immune deficiency, chronic basic diseases, and severe enteritis need antibiotics (Nair et al., 2021). However, with the wide application of antibiotics, the drug resistance rate is increasing yearly, which poses a severe challenge to treating *Salmonella* infection. The rational selection and use of antibiotics have become important issues worldwide.

Ampicillin, chloramphenicol and cotrimoxazole were the first-line antibiotics for the treatment of salmonellosis, resistance to first-line antibiotics used to treat infections caused by *Salmonella* is increasing. The emergence and spread of multi-drug resistance (MDR) pose a major threat to the effective treatment and control of Salmonellosis, macrolides(such as azithromycin) and carbapenems have been designated as the most important antibiotics for the treatment of *Salmonella* disease (Carey et al., 2021).

Azithromycin is the only remaining oral drug for the treatment of extensively drug resistant (XDR) *Salmonella* infection (Plumb et al., 2019). Particularly noteworthy is the emerging resistance to azithromycin, which will cause people to worry about incurable infection. It is necessary to monitor and diagnose azithromycin resistance to guide rational use and prevent the prevalence and expansion of drug resistance.It was reported that the azithromycin resistance rate of NTS isolates from Taiwan (3.1%) is much higher than that of NTS isolates from European countries and the United States (Chiou et al., 2023).Antimicrobial resistance is a major public health problem in the world, and the mechanism of azithromycin resistance is rarely studied.

This study aimed to determine the azithromycin resistance genes and plasmids of *Salmonella* enterica isolates from children attending the Shenzhen Children’s Hospital by susceptibility testing and whole genome sequencing (WGS), and provide information of monitoring and periodic review of sensitivity data to ensure the adequacy of treatment guidelines.

## 2 Materials and methods

### 2.1 Ethics approval and consent to participate

The data were approved by the ethics committee of Shenzhen Children’s Hospital under document number SEY0132407.

### 2.2 Bacterial collection

*Salmonella* enterica strains were isolated from clinical blood and fecal culture samples collected from the Shenzhen Children’s Hospital between January 2014 and December 2021. The data, including information of children and isolations were collected from Clinical Microbiology Laboratory, Department of clinical Laboratory, We excluded data on contaminated bacteria and duplicate strains detected from the same patient.

### 2.3 Bacterial culture and identification

A BACTEC™ FX-200 automatic blood culture instrument (BD Diagnostic Systems, Sparks, MD, USA) and BacT/Alert 3D blood culture system (BTA3D; bioMerieux, Marcy lEtoile, France) were used for blood culture, 1~10 ml of blood specimens were subjected to blood culture bottles and incubated at 37°C in automated system. Bottles that positive alerts were detected would be removed and samples would be cultured on Columbia blood agar plates and chocolate agar plates. Other samples were cultured and all isolates were identified according to methods of Manual of Clinical Microbiology [11^th^ edition] (James et al., 2015). The bacteria were identified to the genus level using a VITEK 2 COMPACT automatic microbial identification drug sensitivity instrument (Biomerieux, France) and a mass spectrometry system (MALDI-TOF MS, Merier, France), and the serotypes were divided using the Danish Statens Serum Institut diagnostic serum according to structures of somatic O and flagellar H antigens(the Kauffman-White classification).

### 2.4 Drug sensitivity test

The susceptibility of five antimicrobial agents (Oxoid, UK), including ampicillin (AMP), ceftriaxone (CRO), chloramphenicol (CL), trimethoprim-sulfamethoxazole (SXT), ciprofloxacin (CIP), and azithromycin (AZM), was determined using the disk diffusion method to screen azithromycin resistant strains. The MIC value of azithromycin resistant stains were calculated using the E-test method (Biomerieux,France). The results, evaluated according to the judgment results of the breaking point standards recommended by the Clinical and Laboratory Standards Institute (CLSI) M100 2021 (Clinical and Laboratory Standards Institute[CLSI], 2021), were divided into sensitivity, mediation, and drug resistance. Since there is no definite azithromycin CLSI break point for any *Salmonella* serotype except *S.*Typhi, the azithromycin resistance standard of *S.* Typhi was used, i.e., inhibition zone ≤ 12 mm and MIC ≥ 32 mg/mL was determined as drug resistance.

### 2.5 Whole-genome sequencing

The *S.* enterica isolates for azithromycin resistance were further subjected to whole-genome sequencing. Genomic DNA was extracted from overnight cultures using a QIAamp DNA mini kit (Qiagen) according to the manufacturer’s instructions. DNA quality was assessed using Nanodrop spectrophotometry (Thermo Fisher), and quantity was assessed using the Qubit 4.0 system (Thermo Fisher). The DNA libraries were constructed with 150-bp paired-end whole-genome sequencing using the Illumina HiSeq 2500 system (Huada, Shenzhen, China) (Ma et al., 2020). The obtained paired-end Illumina reads were assembled *de novo* using SPAdes v3.6.2 (default parameters except -careful and -k 21,33,55,77,99,127). In addition, to obtain long read sequences, selected strains were further sequenced using Oxford Nanopore MinION flowcell R9.4 (Li et al., 2018). *De novo* hybrid assembly was performed using a combined Illumina HiSeq and Nanopore sequencing approach (Nextomics). Genome assembly was performed with Unicycler version 0.4.1 using a combination of short and long reads, followed by error correction with Pilon version1.12 (Wick et al., 2017) (Walker et al., 2014).

### 2.6 Data analysis

An in silico multilocus sequence typing (MLST) scheme was used to subtype the isolates using mlst software (version 2.19.0) (Larsen et al., 2012). The chromosome and plasmid sequences were annotated using the prokaryotic gene prediction tool Prokka (Seemann, 2014). The plasmid incompatibility type was searched using the online tool PlasmidFinder (https://cge.cbs.dtu.dk//services/PlasmidFinder/) (Carattoli et al., 2014). Antibiotic resistance genes were identified using both the Comprehensive Antibiotic Resistance Database (CARD) database (Alcock et al., 2020). Comparative plasmid illustration was implemented by BRIG (http://brig.sourceforge.net) (Alikhan et al., 2011). BLAST (https://blast.ncbi.nlm.nih.gov/Blast.cgi) was used for comparative analysis through coverage and identities (Camacho et al., 2009).

Genomic sequences and the associated metadata of 10561 *Salmonella* strains stored in the NCBI GenBank database were obtained. Bacterial core genome single nucleotide polymorphism (cgSNP) analysis between 15 azithromycin-resistant clinical isolates and 10561 complete or draft genomic sequences of *Salmonella* enterica strains was performed to construct a maximum likelihood phylogenetic tree using Parsnp software (Kaas et al., 2014).This analysis was performed using the default parameters, and *S.* enterica subsp. enterica serovar Typhimurium str. LT2 (RefSeq ID: NC_003197.2) as the reference genome. Phylogenetic trees were visualized and annotated by the Interactive Tree of Life (iTOL) V5 web server (Letunic and Bork, 2021).

### 2.7 Statistical analysis

We adopted WHONET 5.6 software for data analysis, and the comparison of rates adopts χ (2) inspections.

## 3 Results

### 3.1 Clinical informations

A total of 15 *Salmonella* strains were detected in 13 children. If the time interval between the detection of *Salmonella* in a child exceeded three days, it was collected as a new strain and tested accordingly. The clinical, demographic, and laboratory characteristics of the 13 patients with azithromycin-resistant Salmonella infections are displayed in **Table 1**.

**Table 1:** General information and clinical features of pediatric patients with azithromycin-resistant *Salmonella enteric*.

Characteristics	Number (n=13)
Age, in median months	14 [3,116]
Sex	
Male	9
Female	4
Underlying diseases	1
Clinical symptoms	
Fever 37.4-39°C	3
39.1-40 °C	9
>40.1 °C	1
Abdominal pain and diarrhea	13
anemia	3
Laboratory findings	
Total leucocyte count, ×10^9^ cells/L	11.15 [3.67, 33.10]
Lymphocyte count, ×10^9^ cells/L	3.37 [1.86, 6.56]
Eosinophil count, ×10^9^ cells/L	0.11 [0.00, 0.74]
C-reactive protein mg/L	23.47 [2.34, 92.50]
Aspartate aminotransferase IU/L	27.0 [12.0, 38.0]
Course of disease, days	5 [3,14]
Clinical Outcomes	
Cure	13

### 3.2 Serotypes of azithromycin-resistant *S.* enterica

**Figure 1:**
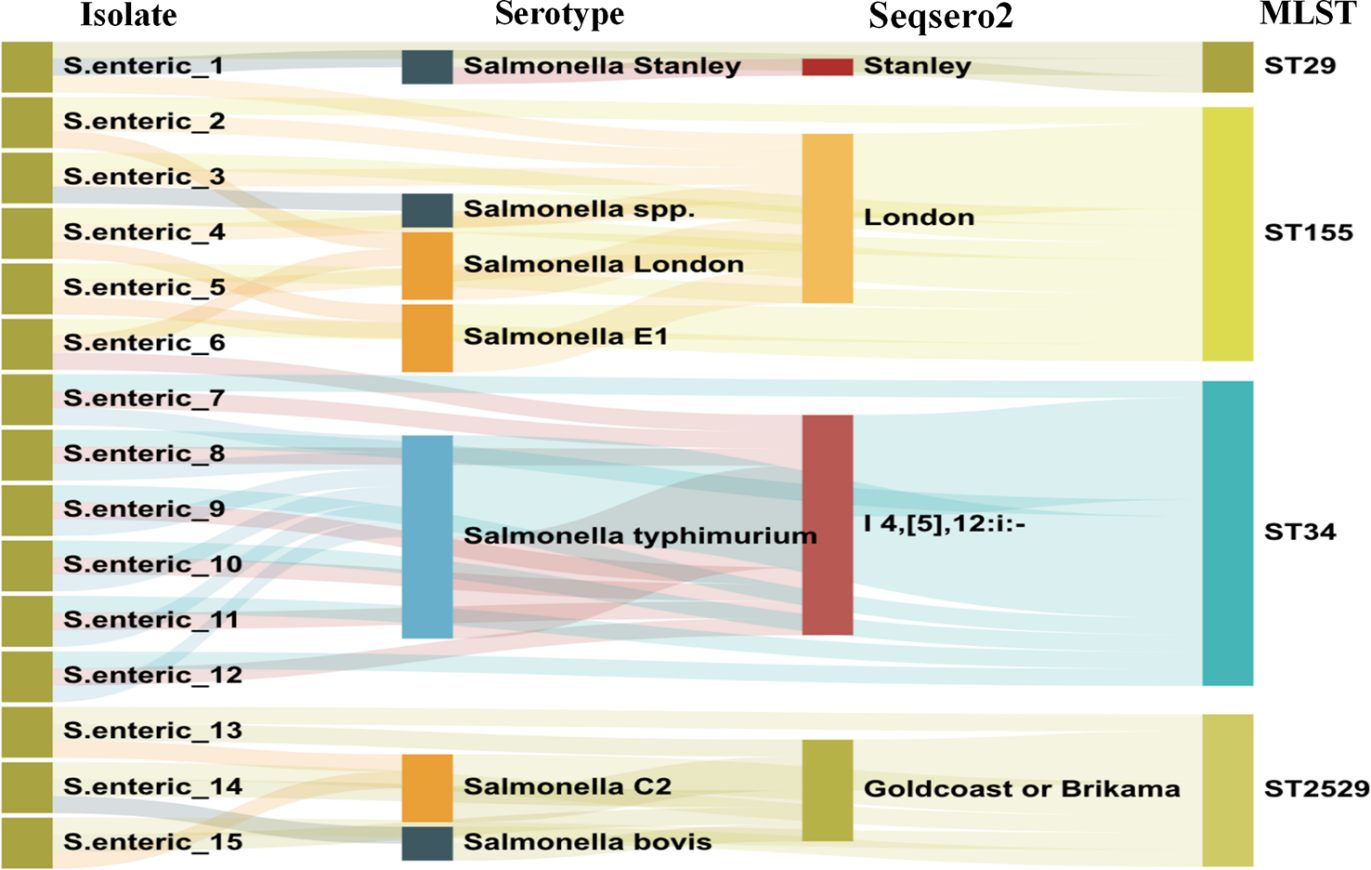
Serotype and MLST type distribution of 15 azithromycin resistant Salmonella strains.

After routine drug sensitivity test screening of 487 retained *S.* enterica strains, 15 azithromycin-resistant *S.* enteric strains were detected. The serotype distribution was identified using traditional *Salmonella* serum, and the results of the data analysis after WGS and assembly are displayed in **Figure 1**. It was demonstrated four serotypes in the 15 *Salmonella* strains, including *S*.Stanley, *S*.London, *S*.Goldcoast or Brikama and *S*.I 4,[5],12:i:-.

### 3.3 Antimicrobial susceptibility profiles

Testing of the susceptibility of 15 *Salmonella* isolates (**Table 2**) to 6 antibiotics. Among these isolates, 100% (16/16) were resistant to AZM, while all isolates were also resistant to AMP. The resistance rates against SMZ, CL, CRO, and CIP were 87.3%, 80.0%, 46.7%, and 20.0%, respectively. For AZM, 26.67% of the isolates showed the highest MICs of 256 µg/mL, 20.0% showed MICs of 64 µg/mL, and 53.33% showed MICs of 32 µg/mL.

**Table 2:** Distribution of drug sensitivity and MIC value of azithromycin resistant *S*. enterica.

	Disk Diffusion Test						E-TEST
Isolate	AMP	CRO	SXT	CIP	CL	AZM	AZM
							(MIC, µg/mL)
S.enteric 1	R	S	R	I	R	R	256
S.enteric 2	R	S	R	I	R	R	32
S.enteric 3	R	R	R	I	R	R	32
S.enteric 4	R	S	R	I	R	R	32
S.enteric 5	R	S	R	R	R	R	32
S.enteric 6	R	S	R	I	R	R	32
S.enteric 7	R	R	S	S	S	R	256
S.enteric 8	R	R	R	R	R	R	32
S.enteric 9	R	R	R	R	R	R	32
S.enteric 10	R	R	S	I	R	R	32
S.enteric 11	R	R	R	I	R	R	256
S.enteric 12	R	R	R	I	R	R	256
S.enteric 13	R	S	R	I	S	R	64
S.enteric 14	R	S	R	I	R	R	64
S.enteric 15	R	S	R	I	S	R	64
Resistant (%)	100	46.7	86.7	20	80	100	
Intermediate (%)	0	0	0	73.3	0	0	
Sensitivity (%)	0	53.3	13.3	2.2	20	0	

The bold values of 'R" means "Resistance", "I" means " Intermediate" and "S" means " Sensitive".

### 3.4 Phylogenetic analyses

**Figure 2:**
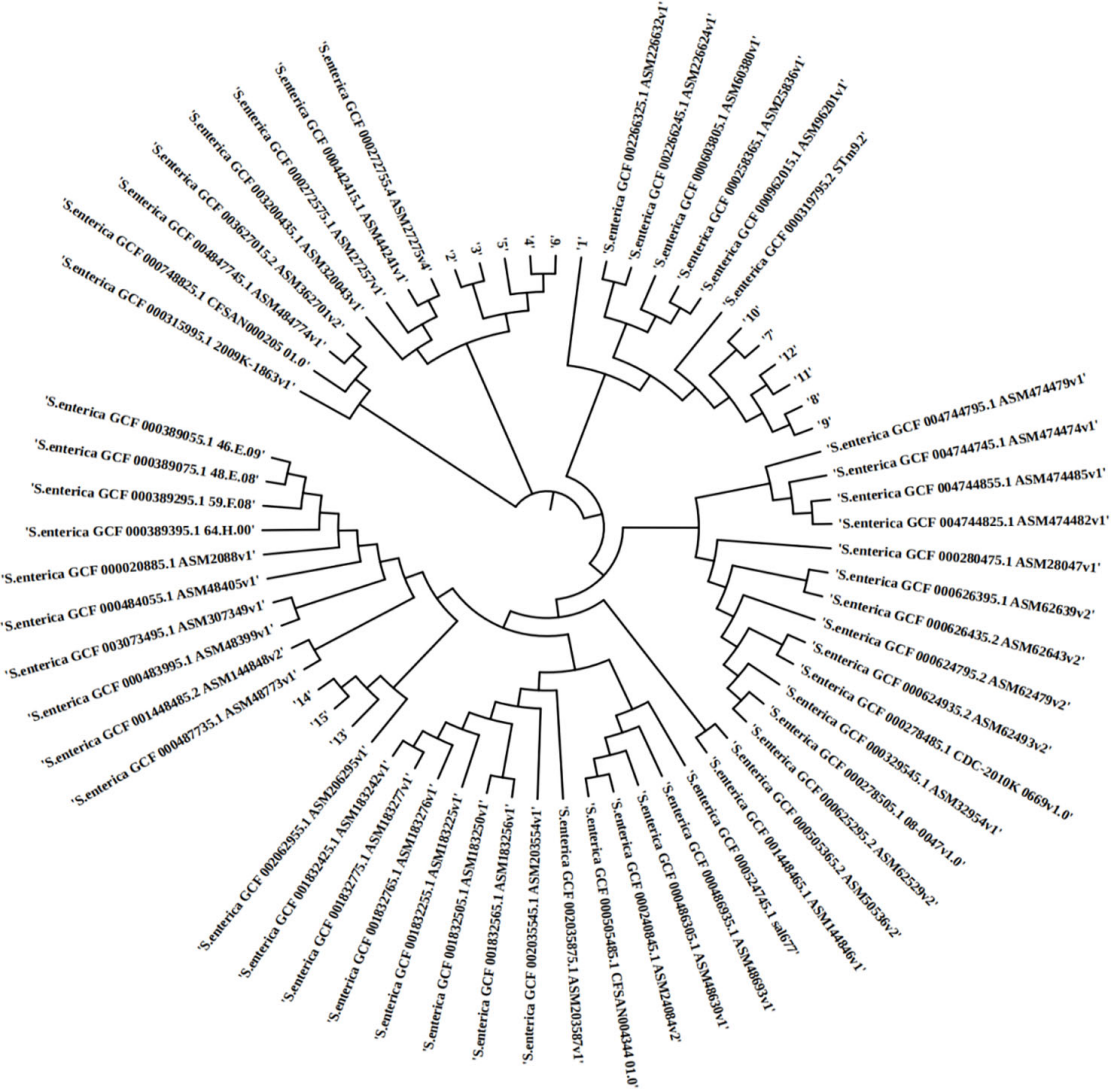
The phylopene relationship between 15 *Salmonella* enterica isolates and a total of 68 *Salmonella* enterica strains currently deposited in the NCBI GenBank database. The distance of SNPSs is represented by the branch length.

In silico MLST analysis indicated that 15 isolates represented four sequence types, which were assigned to ST 29 (1/16), ST 34 (6/15), ST 155 (5/15), and ST 2529 (3/16) (**Figure 1**). We performed phylogenetic analysis of 15 *S.* enteric*a* isolates and generated a phylogenetic tree with 68 strains and 3,067 SNP loci (**Figure 2**). The phylogenetic tree showed that the 15 self-tested strains clustered and formed four independent branches. The predominant ST type of the clade containing five samples is ST34; however, sample 1 is the rare ST type ST 29. The ST types of clades containing four and three samples were ST 155 and ST 2529, respectively. According to the results of phylogenetic analysis, the closest relative of ST34 isolates was identified in 2007 from a fecal sample in Australia, and the closest relative of ST34 isolates was identified in 2010 from a poultry small intenstine in Nigeria.

### 3.5 Genotypic characterization of antimicrobial resistance

**Figure 3:**
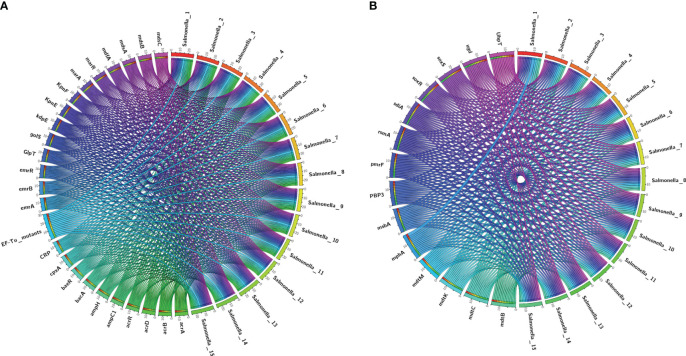
Detection of ARGs over the 15 plates. **(A, B)** Chord diagram illustrating the correlations between ARGs and the ARG-carrying pathogenic species. Thickness of the lines represents the number of samples observing such correlation. A total of 39 ARGs were shared by the 15 isolates.

**Figure 4:**
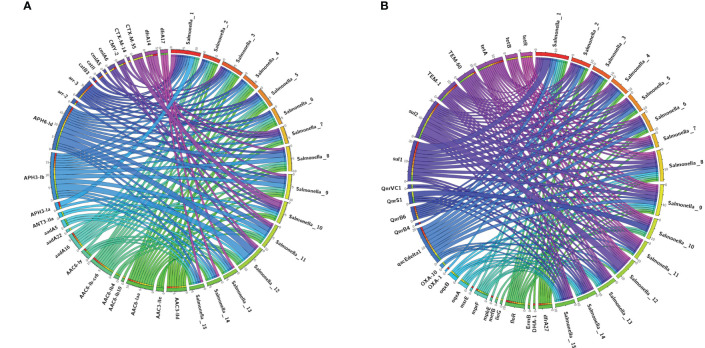
The upstream and downstream parts of the mphA resitance gene fragments shared the same backbone sequence containing genes.

We performed antimicrobial resistance gene analysis on 15 strains, which were extracted from whole genome sequencing (WGS) analysis. A total of 91 ARGs were found in the 15 isolates (**Figures 3**, **4**), of which 39 ARGs were shared by the 15 isolates (**Figure 3**). The results shown in **Figure 3** indicate that the 15 self-tested azithromycin-resistant strains all carried the azithromycin resistance gene *mphA* (**Figure 3B**), and *the S.* enterica*_*1 isolate carried two *mphA* genes. *S.* enterica*_*7 also carried the other azithromycin resistance gene *ErmB*. In addition, some CTX-M-type extended-spectrum beta-lactamase (ESBL) genes *(blaCTX-M-14, blaCTX-M-55*) and aminoglycoside resistance genes (*APH(3’)-Ia, APH(3’’)-Ib*, and *APH(6)-Id*) were identified among these isolates. Additional *AMR* genes (*emr* family genes, *dfrA* family genes and *sul* family) were also identified among these isolates. Overall, phenotypic resistance was highly correlated with the presence of known resistance determinants.

### 3.6 Genetic characterization of *mphA*-carrying plasmid

**Figure 5:**
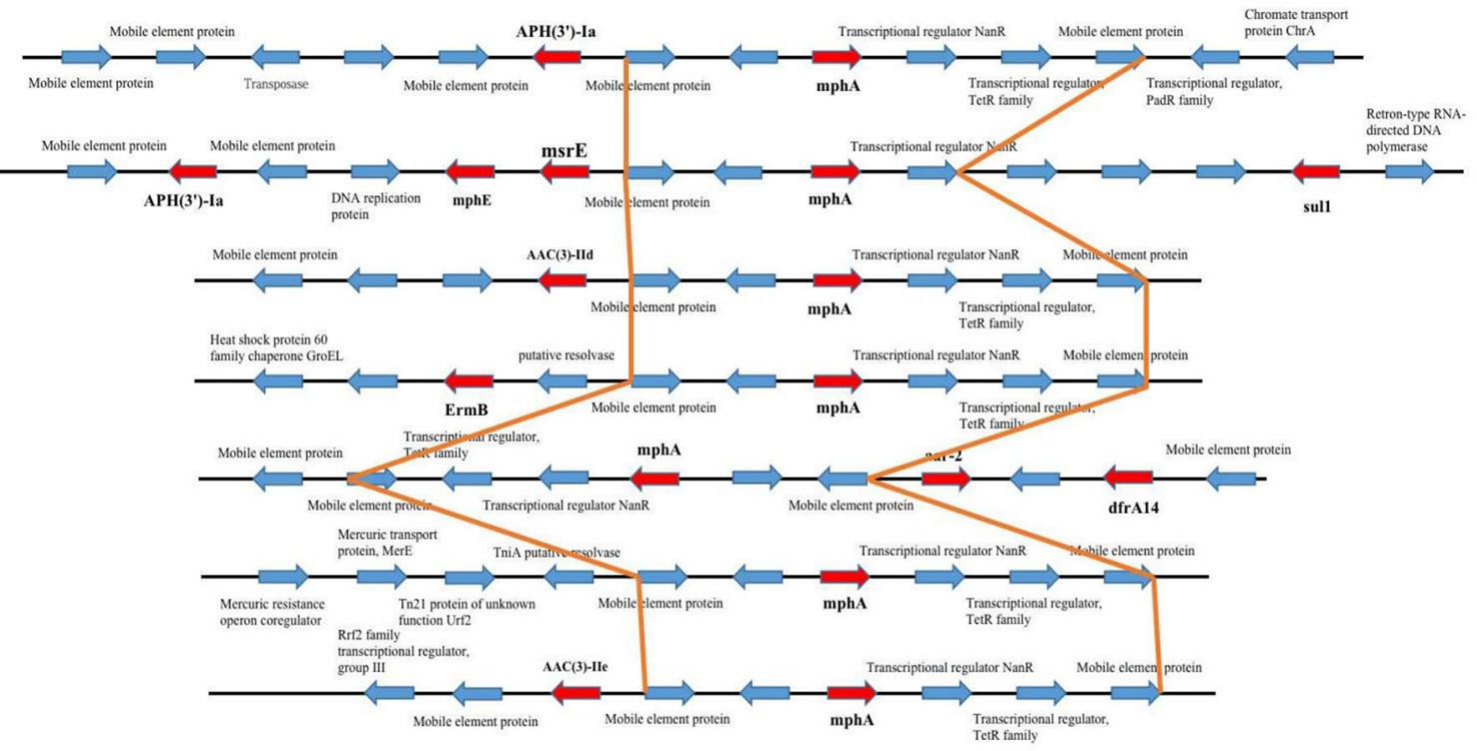
mph(A) were located on two different plasmids in S. enteric-7. **(A)** The large plasmid. **(B)** The small plasmid.

Among the 15 isolates (S1-S15) with complete genome sequences, *mphA* was positive in all isolates and was located on several different plasmids。The upstream and downstream parts of the *mphA* resistance gene fragments shared the same backbone sequence containing genes for 2 mobile element protein genes, transcriptional regulator (*TetR* family), transcriptional regulator *NanR*, and the genetic structure of the *mphA* gene (**Figure 5**). Blast alignment showed that stable sequences containing *mphA* appeared in multiple plasmid structures, and the sequence identity was 100%.

**Figure 6:**
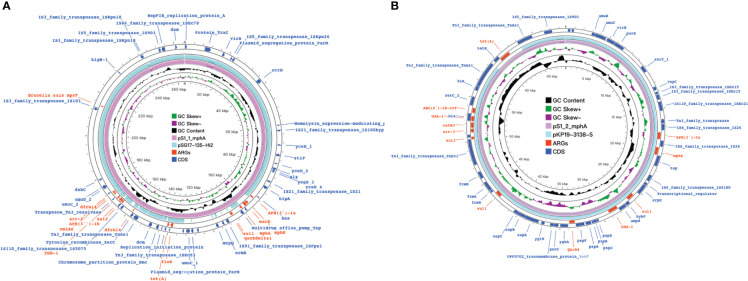
mph(A) were located on two different plasmids in S. enteric-2 (also include S.enteric-3, 4, 5, 6) and S. enteric-7. **(A)** The plasmid in S.enteric-2 (also include S.enteric-3, 4, 5, 6) **(B)** The plasmid in S.enteric-7.

**Figure 7:**
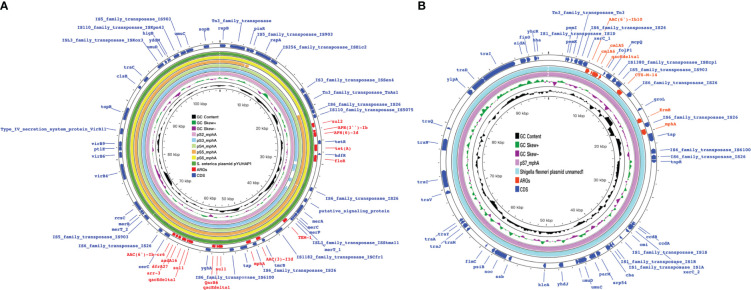
mph(A) were located on two different plasmids in S. enteric-8(also include S.enteric-9) and S.enteric-10. **(A)** The plasmid in S. enteric-8 (also include S. enteric-9) **(B)** The plasmid in S.enteric-10.

**Figure 8:**
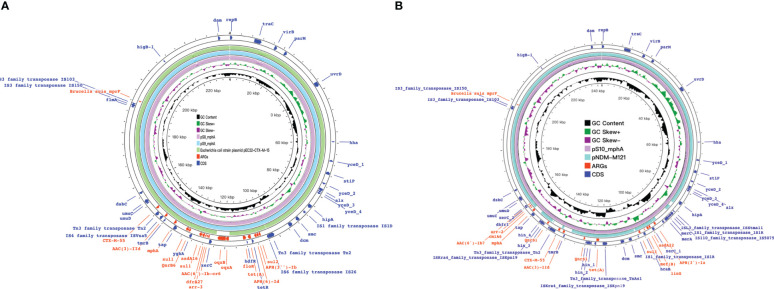
Detection of ARGs over the 15 isplates. **(A, B)** Chord diagram illustrating the correlations between ARGs and the ARG-carying pathogenic species. Thickness of the lines represents the number of samples observing such correlation. A total of 52 ARGs were not shared by the 15 isolates.

**Figure 9:**
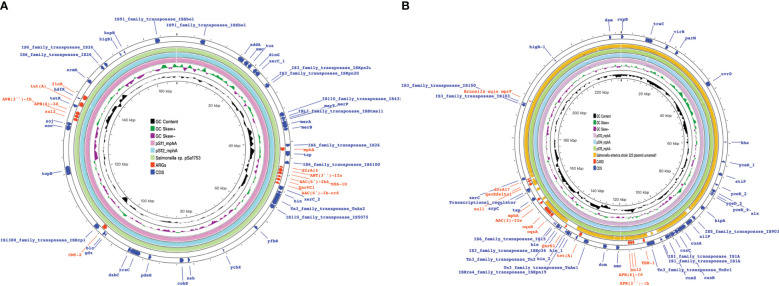
mph(A) were located on two different plasmids in S. enteric-11(also include S. enteric-12) and S.enteric-13(also include S.enteric-14, 15) **(A)** The plasmid in S.enteric-11(also include S.enteric-12) **(B)** S.enteric-13(also include S.enteric-14, 15).

We used Blastn to compare the plasmid sequence in this study with that on the NCBI website, to find the closest homologous plasmid, the results are shown in the **Table 3**. Because the plasmid is not species-specific, lead to cannot form a complete phylogenetic tree containing all the plasmids in this study. Among the 15 isolates (S1-S15), we detected 8 diffrenet plasmids, and found cloest homologous plasmids to these eight plasmids(>82% coverage, >99.9% nucleotide sequence identity). 4 plasmids are homologous with plasmids from *Salmonella* spp., 2 plasmids are homologous with plasmids from *Escherichia coli*, 1 plasmid is homologous with plasmid from *Klebsiella pneumoniae*, and 1 plasmid is homologous with plasmid from *Shigella flexneri*. The plasmid carried is closely related to the phylogenetic aggregation of the strains. Strains with close branches carry the same plasmid, such as, strain S2-S6, strain S8-S9, strain S11-S12, strain S13-S15 (**Figures 6**–**9**).

**Table 3:** NCBI retrieval of homologous plasmids.

Strian	Plasmid	Homologous plasmid	Host bacteria	Query Cover(%)	Per. ident(%)
*S*. enterica1	pS1_1	pSG17-135-HI2	*S*. enterica subsp. enterica serovar Agona strain SG17-135	82%	99.99
*S*. enterica1	pS1-2	pKP19-3138-5	*Klebsiella pneumoniae* strain KP19-3138	94%	99.97
*S*. enterica2	pS2	pYUHAP1	*S*. enterica subsp. enterica serovar London strain HA3-IN1	100%	99.97
*S*. enterica3	pS3	pYUHAP1	*S*. enterica subsp. enterica serovar London strain HA3-IN1	100%	99.97
*S*. enterica4	pS4	pYUHAP1	*S*. enterica subsp. enterica serovar London strain HA3-IN1	100%	100
*S*. enterica5	pS5	pYUHAP1	*S*. enterica subsp. enterica serovar London strain HA3-IN1	100%	99.95
*S*. enterica6	pS6	pYUHAP1	*S*. enterica subsp. enterica serovar London strain HA3-IN1	100%	99.97
*S*. enterica7	pS7	unnamed1	*Shigella flexneri* strain STLEFF_34	100%	99.99
*S*. enterica8	pS8	pEC22-CTX-M-15	*Escherichia coli* strain EC20	97%	99.99
*S*. enterica9	pS9	pEC22-CTX-M-15	*Escherichia coli* strain EC20	97%	99.99
*S*. enterica10	pS10	pNDM-M121	*Escherichia coli* strain ECNB21-M121	89%	99.9
*S*. enterica11	pS11	pSa1753	Salmonella sp. strain Sa1735	100%	99.99
*S*. enterica12	pS12	pSa1753	Salmonella sp. strain Sa1735	100%	99.99
*S*. enterica13	pS13	unnamed1	*S*. enterica subsp. enterica serovar Indiana strain 222	90%	99.98
*S*. enterica14	pS14	unnamed1	*S*. enterica subsp. enterica serovar Indiana strain 222	92%	99.97
*S*. enterica15	pS15	unnamed1	*S*. enterica subsp. enterica serovar Indiana strain 222	92%	99.98

The plasmids integrating multiple functional mobile elements and azithromycin resistance genes, which can be transferred without antibiotic selection pressure. Our plasmids sequence analysis indicates that the *mphA*-bearing *IncX1* plasmids were hypothetically mobilizable and could move into the chromosome *via* insertion sequences such as *IS21/IS26/ISVsa5/IS15*. The typical *IS26-mphA-tap* transposition unit was embedded in the *IncX1* plasmid and other MDR plasmids, such as *IncHI2* (**Figures 6**, **7**, **9**). This highlights the pivotal role of *IS26* and tap in the transmission of *mphA* among plasmids and chromosomes. Detailed analysis of *mphA*-bearing contigs in the 15 *mphA*-positive isolates showed that the core structure *IS-mphA-tap* (n = 15) and seven additional core structures were prevalent among these isolates (**Figures 6**–**9**). However, the complete structures around *IS-mphA-tap* were identified because of short and long fragmented assembled contigs based on Illumina short-read data and Nanopore long-read data.

## 4 Discussion

Azithromycin was discovered in 1980 by the Yugoslav pharmaceutical company Pliva and was on the List of Essential Medicines of World Health Organization’s List (Wang et al., 2020).

Similar to other macrolides, azithromycin mainly targets the P site of the ribosomal 50S subunit, one of the most conserved biomolecules in bacteria. At present, research on the mechanism of azithromycin resistance is increasing. Various reports on azithromycin-resistant *Salmonella* associated with *mphA* found that macrolide-2’-phosphotransferase encoded by *mphA* can mediate the increased resistance of NTS to azithromycin (Centers for Disease Control and Prevention(CDC)). In 2016, Nair Satheesh used WGS and drug resistance phenotype identification to determine the potential mechanism of azithromycin resistance of *S.*enteritidis isolates in the UK. Among 685 strains, 15 were resistant to azithromycin, and 12 encoded *mphA (* Nair et al., 2016). *mphA*-mediated azithromycin resistance has been reported in China.Among the 32 strains of azithromycin-resistant *Salmonella typhimurium* collected in Shanghai by Wang J et al (Wang et al, 2017).15 strains had MIC ≥ 128 μg/mL, from which *mphA* was detected.Hong et al. in Taiwan found that *mphA* existed in azithromycin-resistant *Salmonella* Typhi by identifying the drug resistance genes of serotypes of five strains from humans, pigs, and chickens (Hong et al., 2018). The emergence of *mphA* and its horizontal transmission ability therefore threaten the use of azithromycin for *Salmonella* infection. However, the mechanism of *mphA* level transfer is still unclear and needs to be further clarified using molecular epidemiology and comparative genomics.

This study demonstrated that *mphA* was detected in 15 azithromycin-resistant *Salmonella* strains, with no other azithromycin-related drug resistance gene detected, suggesting that the drug resistance gene is prevalent in Shenzhen Children’s Hospital. Among the 15 azithromycin-resistant *Salmonella* strains detected in 13 children, only one child had the basic disease (after neuroblastoma surgery) and was treated with special grade antibiotics (meropenem). One case was complicated with adenovirus infection with severe diarrhea symptoms. The antibiotics used were all third-generation cephalosporins. The treatment effect was good, and the patient was cured and discharged.

In this study, the correlation of the MIC with a resistance gene showed that the MIC ranged between 32 and 256 µg/mL among the study isolates and that the *mphA* gene was found in all *S.* enterica isolates. There are also reports of E. coli isolates with an MIC of ≥256 µg/mL carrying the *mphA* gene, followed by *ermB* and *mphB* in isolates with an MIC of >1024 and 128 µg/mL, respectively (Phuc Nguyen et al., 2009).

Generally, azithromycin-resistance genes such as *mphA* and *ermB* were reported to be carried in plasmids (Darton et al., 2018).Remarkably, in this study, the plasmid carrying the *mphA* gene was found to be carried in 8 different plasmids, which might be possible due to the presence of various insertion sequences and other mobile elements in *S*. enterica. Apart from this unique finding, genome analysis revealed the presence of multiple resistance genes that were expected. The genome also contained various mobile genetic elements that are reported to play a significant role in AMR dissemination in *S.* enterica (Yu et al., 2012).

An earlier study by Cho S showed that *E.* coli acts as a reservoir for macrolide-resistance genes from which resistant *S.* enterica might have emerged through horizontal gene transfer (Cho et al., 2019). This phenomenon has been previously demonstrated with E. coli donating *mphA* to *S.* sonnei (Phuc Nguyen et al., 2009). In this study, we looked for the occurrence of a similar event among the studied isolates. We compared the plasmid profile carried by *S.* enterica and *E. coli* carrying the macrolide resistance gene to identify the backbone similarity. Although the analysis revealed several genes in common, the *S.* enterica plasmid harbored an additional tra operon compared to *E.coli*, which might have been acquired due to evolution over time. The *tra* genes have always been the only genomic factors that make the plasmid conjugative. Interestingly, Benz et al. showed that plasmid transfer is mainly based on functional *tra* (transfer) genes rather than plasmid types (Benz et al., 2021). Furthermore, earlier studies have shown that plasmids carrying required functional *tra* genes can spread even without antibiotic selection pressure. These results highlight the potential risk of plasmids with resistance genes carrying functional *tra* genes being transferred by natural conjugation.

The widespread emergence of MDR *S.* enterica with changing AMR patterns has also been reported. Generally, in *S.* enterica, acquired resistance is more common. β-Lactam resistance is mainly due to the presence of OXA-type β-lactamases, followed by *TEM* and *CTX-M*. Trimethoprim/sulfamethoxazole resistance is encoded by the *dhfr1A* and *sul* genes. Quinolone resistance involves the accumulation of mutations in QRDR and plasmid-mediated quinolone resistance (PMQR) genes. Furthermore, resistance to tetracycline, chloramphenicol and streptomycin has been shown to be due to the presence of *tetA/B*, *catB3* and *aadA* genes or both (Bustamante and Iredell, 2017; Wang et al, 2017; Wang et al., 2018). These results show the ability of species to acquire AMR determinants.

In conclusion, as the extensive resistance of *salmonella* serotypes to conventional first-line drugs, azithromycin have been designated as the most important antibiotics for the treatment of *salmonella*, the novel finding of an integrated plasmid in this study indicates the potential risk of *S.* enterica isolates becoming resistant to azithromycin in the future. Our study highlights the significance of the hybrid assembly approach in complete genome analysis. These findings suggest that it is imperative to monitor *S.* enterica susceptibility and to study the resistance mechanism of *S.* enterica against azithromycin, considering azithromycin is the only remaining oral drug for the treatment of XDR *Salmonella* infection.

## Data availability statement

All clinical isolates sequence data used in the present study has been deposited in the NCBI database under project ID PRJNA879416.

## Author contributions

QW and JD designed the experiments. HW, HC and BH performed the experiments. HW, HC, XH and YC analyzed the data. HW and HC wrote the manuscript. QW, JD, LY and LZ critically commented and revised the manuscript. All authors contributed to the article and approved the submitted version.
